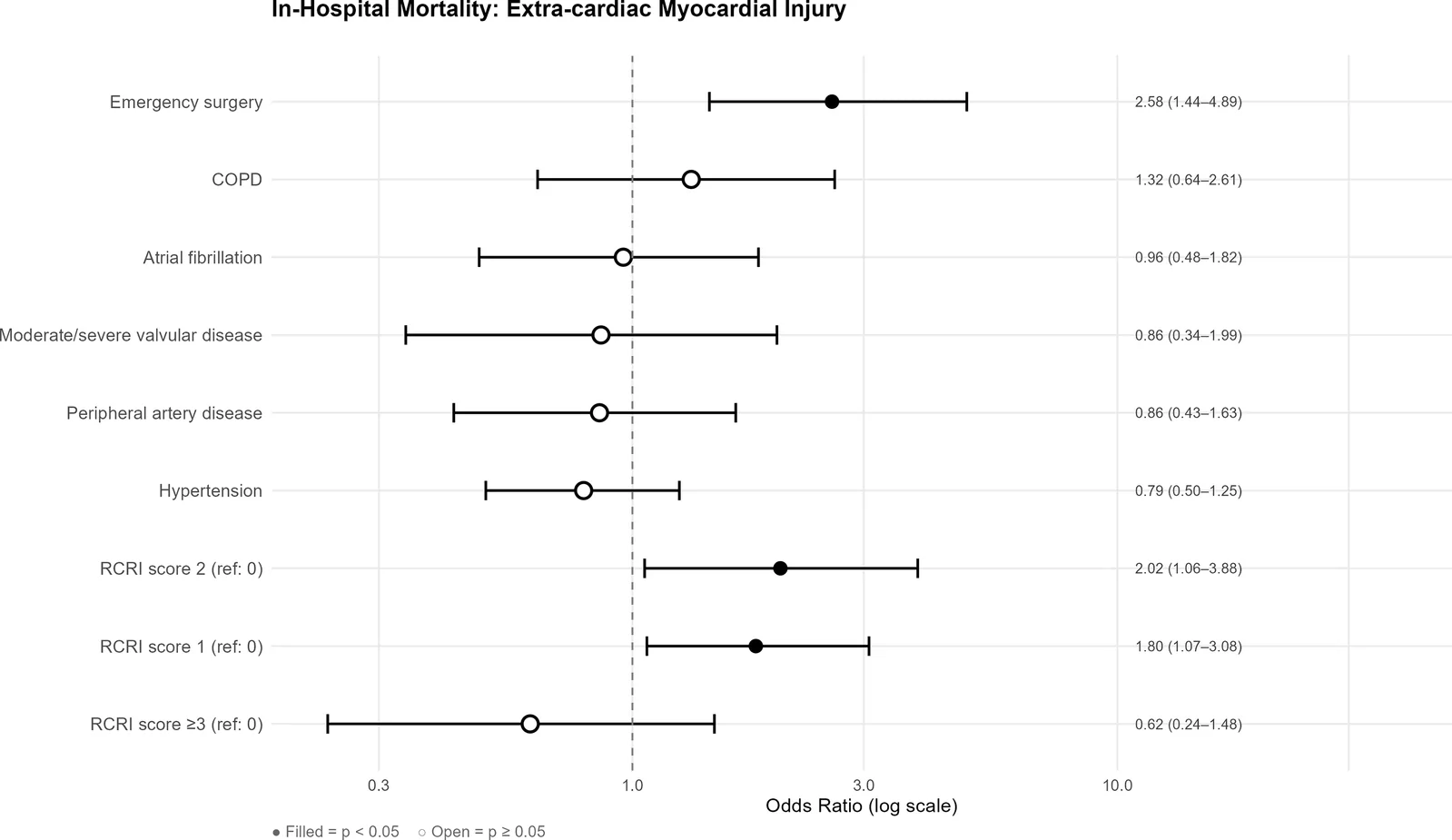# Aetiology and associated outcomes of myocardial injury as defined by postoperative troponin elevation in high-risk patients after noncardiac surgery: a single-centre retrospective cohort study

**DOI:** 10.1016/j.bjao.2026.100647

**Published:** 2026-08-13

**Authors:** Bernard R.B.K. Schockaert, René van Bruchem, Robert Jan Stolker, Mario Stark, Sanne E. Hoeks, Felix van Lier

**Affiliations:** 1 Department of Anaesthesia, Erasmus University Medical Centre, Rotterdam, The Netherlands; 2 Department of Anaesthesia, Pain and Intensive Care, AZ Delta, Roeselare, Belgium

**Keywords:** cardiac troponin, in-hospital mortality, myocardial injury, noncardiac surgery, perioperative outcome

## Abstract

**Background:**

Myocardial injury after noncardiac surgery is common, is frequently clinically silent, and arises from heterogeneous mechanisms. Whether structured aetiological assessment using an existing hierarchical framework is applicable in high-risk patients remains uncertain. The primary aim of this study was to determine the aetiological distribution of myocardial injury in a selectively tested high-risk cohort.

**Methods:**

This single-centre retrospective cohort study included adults undergoing noncardiac surgery during the years 2017 to 2022. Myocardial injury is defined as a high-sensitivity cardiac troponin T concentration >50 ng L^−1^ within 72 h after surgery. Troponin was measured in selected high-risk patients, either routinely or at physician discretion. The primary outcome was aetiological distribution, classified by anaesthetists as extra-cardiac or cardiac, using an existing hierarchical adjudication model. Secondary outcomes were inter-rater agreement after independent reassessment and in-hospital mortality.

**Results:**

Of 6263 patients sampled, 1114 (18%) were diagnosed as having myocardial injury and 954 were analysed. Myocardial injury was classified as extra-cardiac in 544 (57%) and cardiac in 410 patients (43%). Renal failure was the most common extra-cardiac cause (37%) and type 2 myocardial injury the most common cardiac cause (81%). Patients with extra-cardiac injury more often underwent non-elective surgery (78% *vs* 55%; *P*<0.001). Inter-rater agreement after reassessment was 78% (κ=0.55), with discrepancies mainly in type 2 myocardial injury. In-hospital mortality in this high-risk cohort was 20% overall, 24% in those with extra-cardiac injury, and 15% in those with cardiac myocardial injury (*P*=0.002).

**Conclusions:**

In this single-centre high-risk noncardiac surgical cohort, pragmatic aetiological assessment attributed myocardial injury more often to extra-cardiac mechanisms, which were associated with higher in-hospital mortality. Limited by selective postoperative troponin sampling, these findings warrant future research into the prognostic value of troponin elevations across differing aetiologies.

**Editor’s key points**

• Elevated high-sensitivity cardiac troponin T after noncardiac surgery is a marker of myocardial injury and is associated with unfavourable outcomes.
• In this single-centre retrospective report of a selectively tested high-risk noncardiac surgical cohort, anaesthetist-led adjudication attributed myocardial injury, defined based on postoperative troponin concentrations, more often to extra-cardiac than cardiac causes.
• Mortality was higher in the group with an extra-cardiac aetiology of myocardial injury.
• The aetiology of postoperative myocardial injury was heterogeneous, with renal failure being the most common extra-cardiac cause and type 2 myocardial injury (caused by an oxygen supply-demand imbalance not related to CAD) being the most common cardiac cause.

With more than 300 million surgeries performed annually, approximately 4.2 million people die within 30 days after surgery, accounting for 7.7% of global deaths.^1^^,^^2^ In this context, myocardial injury, defined by troponin concentrations exceeding the 99th percentile upper reference limit, has emerged as a prognostic marker.^3^, ^4^, ^5^ In the perioperative setting, typical ischaemic symptoms are frequently absent or attenuated. Consequently, a high index of suspicion is required as elevated cardiac troponin concentrations within 72 h after noncardiac surgery are associated with increased 30-day mortality, even in the absence of ischaemic features.^4^^,^^6^ Hence, several guidelines recommend routine troponin surveillance in patients at risk.^7^, ^8^, ^9^

The underlying aetiology of postoperative troponin elevation is heterogeneous. Several conceptual approaches have therefore been proposed to aid interpretation. The myocardial injury after noncardiac surgery (MINS) construct attributes postoperative troponin elevation to presumed ischaemic cardiac mechanisms after exclusion of non-ischaemic causes such as sepsis, pulmonary embolism, or rapid atrial fibrillation.^10^^,^^11^ An alternative framework is perioperative myocardial injury (PMI), defined as an absolute increase in cardiac troponin concentration from before to after surgery of at least one upper limit of normal.^12^ In a large surgical cohort, PMI was adjudicated according to the presumed most likely aetiology, and mortality differed substantially across aetiological subgroups, with the highest mortality observed in acute heart failure (49%), tachyarrhythmia (40%), and extra-cardiac causes (39%) and the lowest mortality in type 2 myocardial injury (17%).^12^ These findings suggest that aetiological classification may provide clinically relevant information. However, the applicability of this framework, relying on postoperative troponin measurements alone, remains uncertain. Against this background, the primary aim of this study was to determine the aetiological distribution of myocardial injury in a selectively tested high-risk cohort using a pragmatic application of a previously described hierarchical adjudication model.

## Methods

This single-centre retrospective cohort study was conducted at Erasmus University Medical Centre, a tertiary university medical centre in Rotterdam, The Netherlands. The study was conducted in accordance with the Strengthening the Reporting of Observational Studies in Epidemiology guidelines and approved by the local research ethics committee, which waived the requirement for informed consent (MEC-2023-0552; approved 19 October 2023). The study was registered in the Overview of Medical Research in The Netherlands (NL-011210). Data were pseudonymised and stored in a secure digital research environment.

### Study population

Adult patients (≥18 yr) undergoing noncardiac surgery between 2017 and 2022 were included if at least one postoperative high-sensitivity cardiac troponin T (hs-cTnT) concentration exceeded 50 ng L^−1^ within 72 h of surgery. The sample size was based on the available data. In patients with multiple procedures, only the first qualifying operation was analysed. Cardiac surgery, lung transplantation, and day-case surgery were excluded.

### Troponin measurements

Postoperative hs-cTnT was measured routinely after arterial vascular surgery and at the enhanced perioperative care (EPC) unit, a postoperative high-care unit with overnight monitoring for patients at elevated perioperative risk. These groups were considered to be high-risk patients, and therefore, routine postoperative troponin measurement in these settings had been adopted as institutional clinical practice, intended to reflect a pragmatic application of the 2014 European Society of Cardiology (ESC) guideline recommendation applicable during the study period.^13^ Outside these settings, testing was performed at the discretion of the treating clinician. Preoperative troponin measurements were only available in a subset of patients and were therefore not taken into account. Measurements were performed using the Roche Elecsys® hs-cTnT assay (upper reference limit 14 ng L^−1^).

### Data collection

Baseline characteristics were acquired from medical records and consisted of age, sex, type of surgery, and a previous medical history of hypertension, diabetes mellitus with or without insulin use, coronary artery disease, previous myocardial infarction, congestive heart failure, chronic kidney disease, cerebrovascular disease, atrial fibrillation, moderate or severe valvular disease, chronic obstructive pulmonary disease, and peripheral artery disease. Surgery was classified as elective when scheduled in advance; all other procedures, including semi-emergency and emergency operations, were classified as non-elective. Additional perioperative laboratory measurements and admission details were extracted from the institution’s electronic medical record storage database.

### Hierarchical classification of myocardial injury

**Fig 1:**
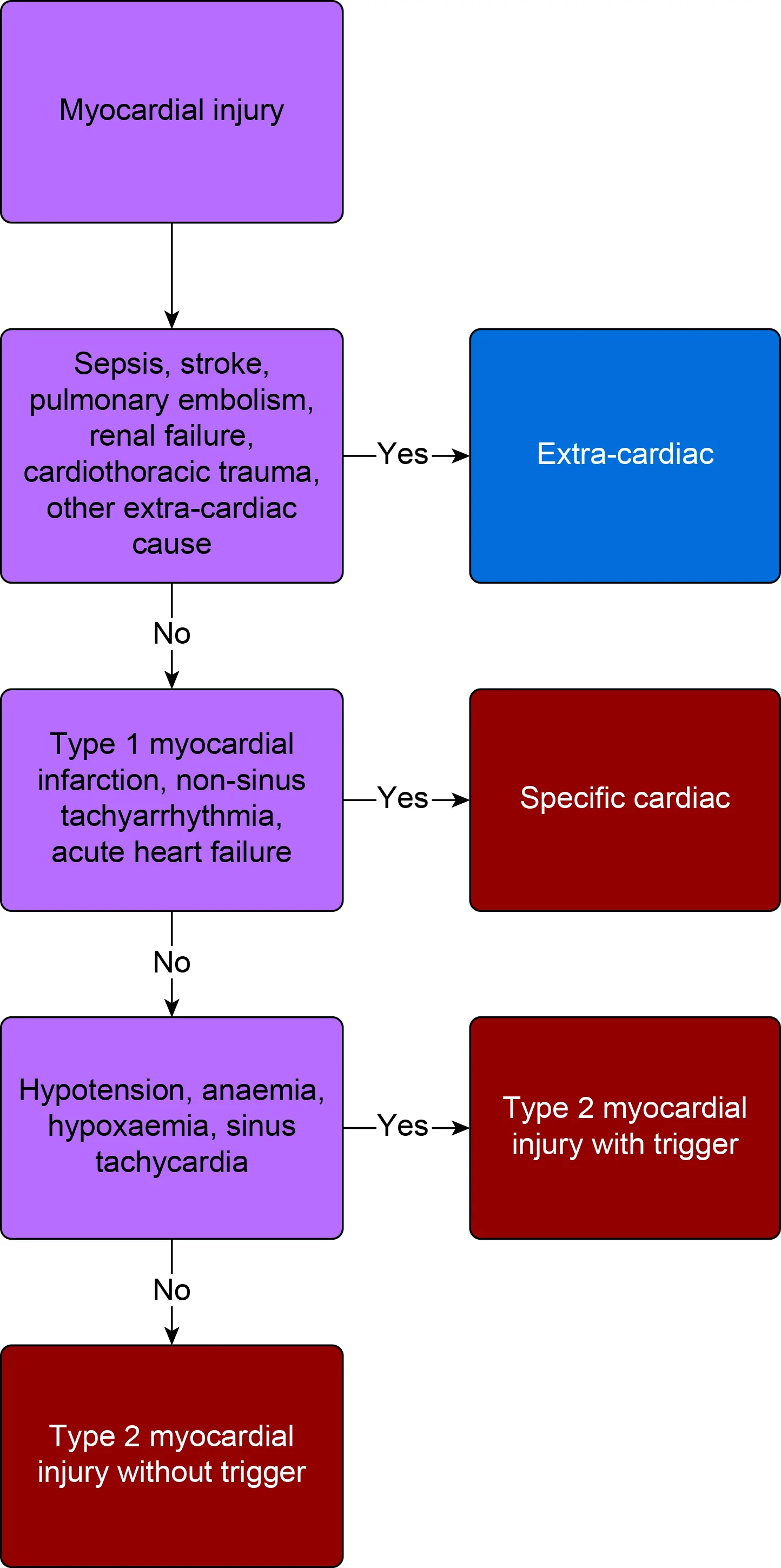
Hierarchical adjudication protocol.

After data extraction, the aetiology of myocardial injury was hierarchically adjudicated based on the patient's context at the time of troponin threshold breach, as documented in the electronic health record, and registered in Castor EDC (Castor Electronic Data Capture, Amsterdam, The Netherlands) using a prespecified hierarchical framework adapted from a previously published classification model (Fig. 1).^12^^,^^14^ In accordance with the original framework, each patient was assigned to one mutually exclusive category representing the single most plausible dominant mechanism, classified as either extra-cardiac or cardiac. Extra-cardiac causes included sepsis, stroke, pulmonary embolism, acute or chronic renal failure, cardiothoracic trauma, and other extra-cardiac causes.^15^ If no extra-cardiac causes were identified, specific cardiac causes were assessed. Cardiac causes comprised type 1 myocardial infarction, non-sinus tachyarrhythmia, or acute heart failure. In the absence of these conditions, myocardial injury was classified as type 2 myocardial injury and further categorised according to the presence or absence of a possible identifiable trigger. Triggers for type 2 myocardial injury included hypotension, hypoxaemia, anaemia, or sinus tachycardia. Patients without an identifiable trigger were classified as having type 2 myocardial injury without a documented precipitant. Applied definitions are presented in Supplement 1.

### Classification and reassessment

The most likely aetiology of myocardial injury was assessed by two independent consultant anaesthetists using the prespecified algorithm described earlier (Fig. 1). Blinded reassessment was performed by two trained anaesthesia-affiliated healthcare professionals using the same protocol. Inter-rater agreement was quantified, and discrepant classifications were retained without consensus resolution and visualised using a Sankey diagram.

### Outcome

The primary outcome was the aetiological distribution of myocardial injury and its classification as extra-cardiac or cardiac. Secondary outcomes included inter-rater agreement after blinded reassessment of myocardial injury aetiology and in-hospital mortality.

### Statistical analysis

All statistical analyses were performed using R version 4.5.2 (R Foundation for Statistical Computing, Vienna, Austria) and RStudio version 2025.09.1 (Posit Software, PBC, Boston, MA, USA). Baseline characteristics of the overall cohort and comparisons between extra-cardiac and cardiac myocardial injury were summarised as counts (%) for categorical variables and compared using Pearson’s χ^2^ test. Continuous variables were assessed for normality visually and using the Shapiro–Wilk test. Normally distributed variables were summarised as mean (standard deviation) and compared using the independent samples *t*-test, whereas non-normally distributed variables were summarised as median [interquartile range] and compared using the Mann–Whitney *U*-test. Two sensitivity analyses were performed: one excluding patients with pre-existing chronic kidney disease and one restricted to hs-cTnT >50 ng L^−1^ measured at the EPC unit or after vascular surgery. Inter-rater agreement for binary myocardial injury classification (extra-cardiac and cardiac) between consultant anaesthetists and anaesthesia-affiliated healthcare professionals was quantified using percentage agreement and Cohen’s κ with 95% confidence interval (CI). In-hospital death and discharge alive within 30 days were analysed as competing events using cumulative incidence functions, with between-group differences assessed using Gray’s test. Separate multivariable logistic regression analyses were performed for extra-cardiac and cardiac myocardial injury, with in-hospital mortality as the dependent variable. Covariates were emergency surgery, Revised Cardiac Risk Index, and baseline characteristics not included in the Revised Cardiac Risk Index.^16^ *P*<0.05 was considered statistically significant.

## Results

**Fig 2:**
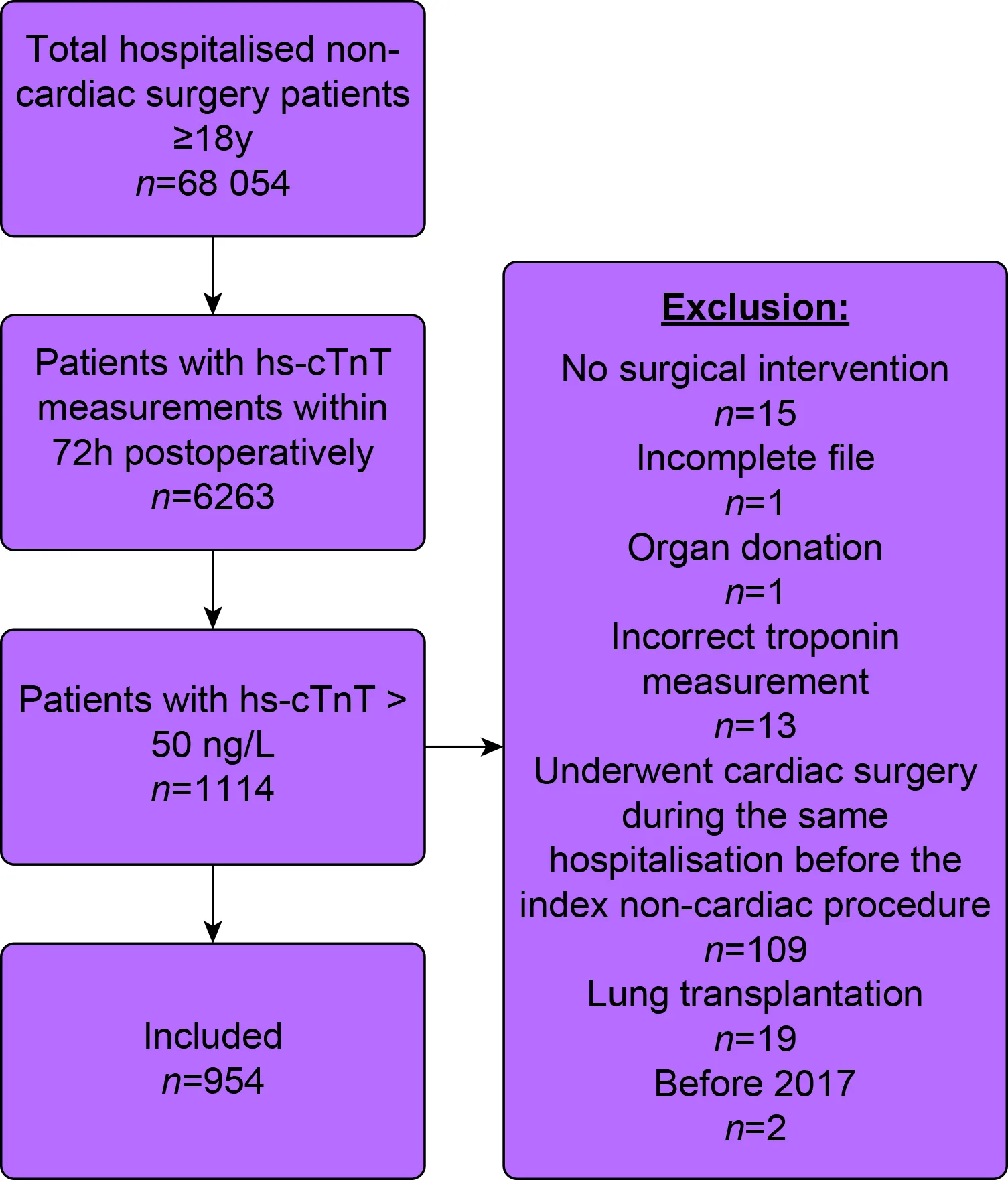
Inclusion and exclusion. hs-cTnT, high-sensitivity cardiac troponin T.

A primary search of the hospital information system identified 68 054 hospitalised adults undergoing noncardiac surgery between 2017 and 2022. Postoperative hs-cTnT was measured within 72 h in 6263 patients; among these, 1114 had hs-cTnT >50 ng L^−1^; 954 patients were eligible for analysis (Fig. 2).

Baseline characteristics of the total study cohort are presented in Table 1. The median age was 66 yr [55–74] and 66% were male; 32% of the procedures were elective. Vascular (19%), general (17%), and neurosurgery (16%) were the most frequent surgical specialties.

**Table 1:** Baseline characteristics. Values are *n* (%) or median [interquartile range]. RCRI, Revised Cardiac Risk Index; TIA, transient ischaemic attack.

	Overall (*N*=954)	Extra-cardiac (*n*=544)	Cardiac (*n*=410)	*P*-value
Age, y	66 [55–74]	63 [51–72]	69 [61–75]	<0.001
Female	329 (34.5)	189 (34.7)	140 (34.1)	0.90
Elective	304 (31.9)	121 (22.3)	183 (44.6)	<0.001
Troponin concentrations in ng L^−1^	109 [69–225]	116 [71–230]	95 [65–206]	0.016
**Surgical specialty**				
Ear-nose-throat	35 (3.7)	9 (1.7)	26 (6.3)	<0.001
General surgery	158 (16.6)	80 (14.7)	78 (19)	0.11
Gynaecology	11 (1.2)	6 (1.1)	5 (1.2)	0.87
Neurosurgery	151 (15.8)	105 (19.3)	46 (11.2)	0.002
Orthopaedics	42 (4.4)	13 (2.4)	29 (7.1)	<0.001
Other	107 (11.2)	57 (10.5)	50 (12.2)	0.43
Thoracic	40 (4.2)	20 (3.7)	20 (4.9)	0.37
Transplant	87 (9.1)	66 (12.1)	21 (5.1)	<0.001
Trauma	113 (11.8)	92 (16.9)	21 (5.1)	<0.001
Urology	28 (2.9)	14 (2.6)	14 (3.4)	0.45
Vascular	182 (19)	82 (15.1)	100 (24.4)	0.001
**Comorbidities**				
Coronary artery disease	217 (22.7)	102 (18.8)	115 (28.0)	0.001
Myocardial infarction	169 (17.7)	83 (15.3)	86 (21.0)	0.027
Peripheral artery disease	183 (19.2)	82 (15.1)	101 (24.6)	<0.001
Stroke/TIA	120 (12.6)	62 (11.4)	58 (14.1)	0.242
Chronic heart failure	107 (11.2)	52 (9.5)	55 (13.4)	0.078
Atrial fibrillation	135 (14.2)	73 (13.4)	62 (15.1)	0.514
Moderate/severe valvular disease	77 (8.1)	38 (7.0)	39 (9.5)	0.194
Diabetes mellitus (non-insulin)	171 (17.9)	95 (17.5)	76 (18.5)	0.732
Diabetes mellitus (insulin-dependent)	80 (8.4)	42 (7.7)	38 (9.3)	0.462
Chronic kidney disease	240 (25.2)	165 (30.3)	75 (18.3)	<0.001
Hypertension	393 (41.2)	208 (38.2)	185 (45.1)	0.038
Chronic obstructive pulmonary disease	116 (12.2)	51 (9.4)	65 (15.9)	0.003
RCRI 0	212 (22.2)	132 (24.3)	80 (19.5)	0.095
RCRI 1	385 (40.4)	221 (40.6)	164 (40)	0.898
RCRI 2	212 (22.2)	110 (20.2)	102 (24.9)	0.102
RCRI ≥3	145 (15.2)	81 (14.9)	64 (15.6)	0.829
**In-hospital mortality**	192 (20)	129 (23.7)	63 (15.4)	0.002

### Classification and reassessment

**Fig 3:**
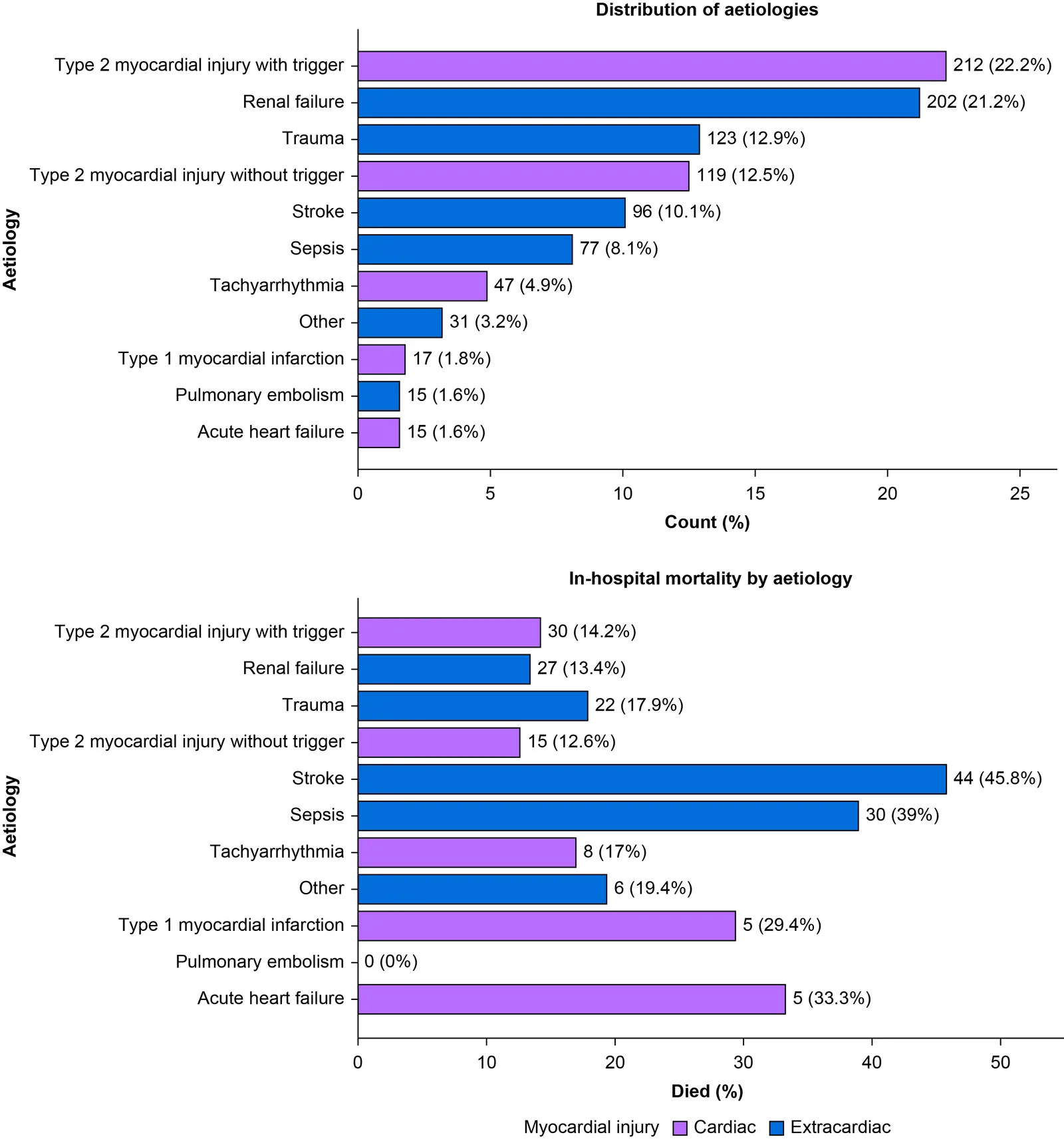
Aetiologies of myocardial injury and in-hospital mortality by aetiology.

Results of the classification process are presented in Fig. 3. Myocardial injury was classified as extra-cardiac in 544 (57%) and cardiac in 410 patients (43%). Baseline characteristics according to myocardial injury aetiology are presented in Table 1. Patients with a cardiac cause were older and more frequently had a history of hypertension, coronary artery disease, myocardial infarction, peripheral arterial disease, and chronic obstructive pulmonary disease than patients with an extra-cardiac cause. Cardiac causes were more common after vascular, ear-nose-throat, and orthopaedic surgery, whereas extra-cardiac myocardial injury occurred more often after non-elective surgery, neurosurgery, trauma, and transplant surgery and in patients with chronic kidney disease.

**Fig 4:**
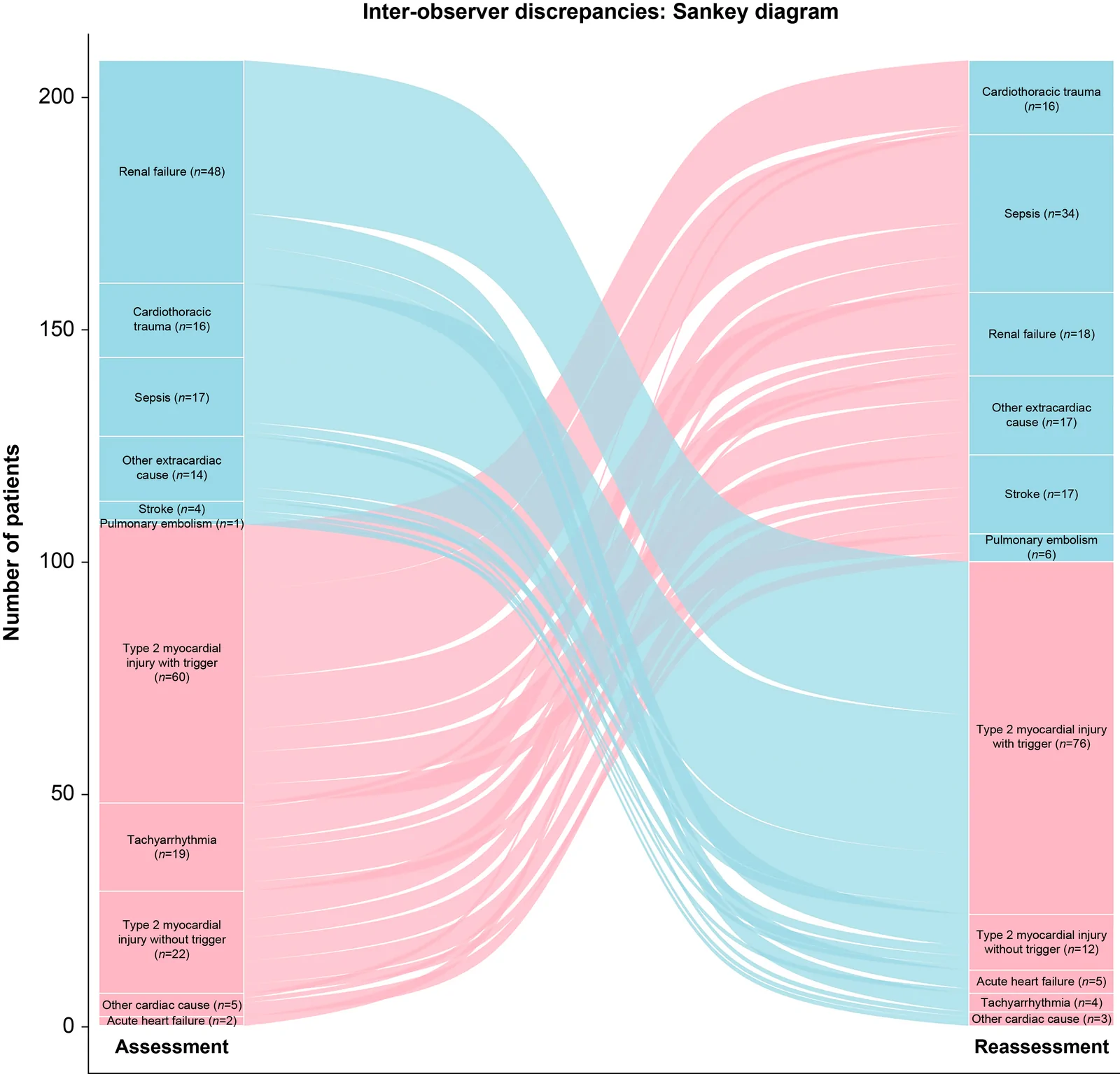
Sankey diagram providing interobserver discrepancies.

Across the overall cohort, the most frequent aetiological categories were type 2 myocardial injury with and without a documented trigger (35%; 22% and 13%, respectively), renal failure (21%), trauma (13%) and stroke (10%) (Fig. 3). Blinded reassessment yielded concordant classification in 739 patients, corresponding to 78% agreement (κ=0.55; 95% CI 0.50–0.60; *P*<0.001). Discrepancies occurred mainly within type 2 myocardial injury (Fig. 4).

### In-hospital mortality

Overall in-hospital mortality in this high-risk cohort was 192 of 954 (20%). Mortality was 129 of 544 (24%) in extra-cardiac causes and 63 of 410 (15%) in cardiac causes (*P*=0.002). In extra-cardiac myocardial injury, the highest mortality was observed in stroke (46%) and sepsis (39%); in cardiac myocardial injury, the highest mortality was observed in acute heart failure (33%) and type 1 myocardial infarction (29%). In type 2 myocardial injury, mortality was 30 of 212 (14%) with a trigger and 15 of 119 (13%) without a trigger (*P*=0.82).

**Fig 5:**
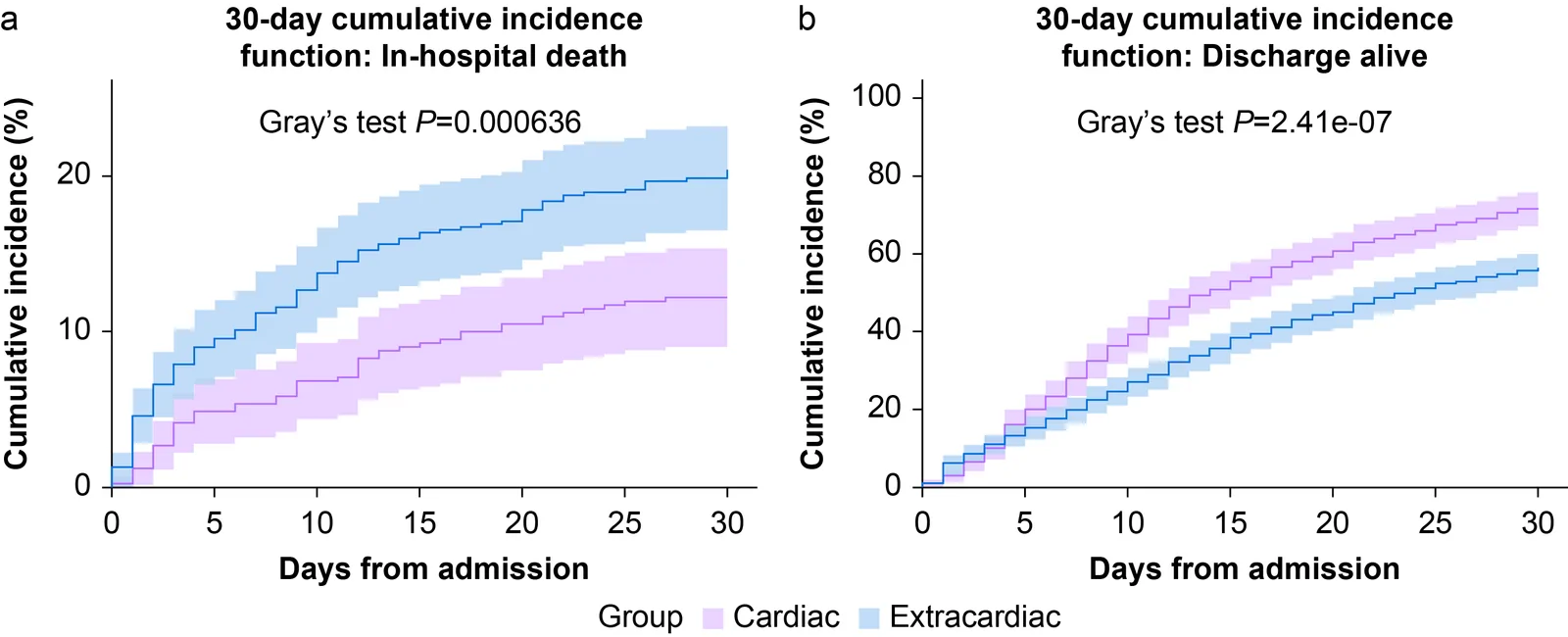
30-day CIF of in-hospital mortality and discharge alive. Shading represents 95% confidence interval. CIF, cumulative incidence function.

When accounting for time to event and discharge alive as competing risk, the 30-day cumulative incidence of in-hospital death was 20% in patients with extra-cardiac myocardial injury and 12% in those with cardiac myocardial injury (Gray’s test *P*<0.001) (Fig. 5). The corresponding cumulative incidence of discharge alive at 30 days was 56% and 72%, respectively (Gray’s test *P*<0.001). Multivariable logistic regression for in-hospital mortality in extra-cardiac and cardiac causes is presented in Supplement 2. Two sensitivity analyses were performed and are presented in Supplement 3: exclusion of patients with pre-existing chronic kidney disease and patients with hs-cTnT >50 ng L^−1^ measured at the enhanced perioperative care unit or after vascular surgery.

## Discussion

In this selectively tested high-risk cohort of 954 patients undergoing noncardiac surgery, myocardial injury was more often attributed to extra-cardiac than to cardiac mechanisms. Extra-cardiac myocardial injury was most commonly attributed to renal failure, trauma, stroke, and sepsis. These patients were younger, underwent more non-elective surgery, and had significantly higher in-hospital mortality than those with cardiac causes.

Within cardiac myocardial injury, type 2 myocardial injury was the predominant type. Owing to the hierarchical classification approach, patients without an identifiable preceding cause were classified in this category by exclusion. No difference in in-hospital mortality was observed between patients with and without a documented trigger. In contrast, type 1 myocardial infarction and acute heart failure were infrequent but associated with relatively high in-hospital mortality. The low incidence of type 1 myocardial infarction is consistent with previous studies indicating that postoperative myocardial infarction is often non-thrombotic.^12^^,^^17^

Similar to the BASEL-PMI investigators, aetiological evaluation at the patient level was performed using a protocolised hierarchical adjudication process.^12^ This approach acknowledged non-ischaemic mechanisms, consistent with Standardised Endpoints in Perioperative Medicine–Core Outcome Measures for Perioperative and Anaesthetic Care and in contrast to the Vascular events In non-cardiac Surgery patIents cOhort evaluatioN (VISION) and American Heart Association MINS construct.^10^^,^^18^, ^19^, ^20^

However, our framework differed in several respects: adjudication was performed by anaesthetists; discrepant classifications were retained deliberately rather than resolved by consensus and inter-rater agreement assessed; renal failure was considered a possible extra-cardiac cause; and an absolute postoperative hs-cTnT threshold >50 ng L^−1^ was used rather than an increase in troponin concentration of at least one upper limit of normal from before to after surgery. This dichotomous threshold has previously shown a rule-in value for non-operative myocardial infarction and prognostic relevance for postoperative long-term outcomes, including in patients with chronic kidney disease.^21^, ^22^, ^23^, ^24^, ^25^ In the sensitivity analysis excluding patients with pre-existing chronic kidney disease, extra-cardiac aetiologies remained predominant and were associated with significantly higher in-hospital mortality, consistent with the primary analysis. In the sensitivity analysis restricted to sampled patients with hs-cTnT >50 ng L^−1^ at the EPC unit or after vascular surgery, extra-cardiac aetiologies also remained more frequent and in-hospital mortality was 12% in the extra-cardiac group and 8% in the cardiac group, although this difference was not statistically significant (*P*=0.263) (Supplement 3). The high overall in-hospital mortality observed in this selected high-risk cohort, particularly in the extra-cardiac group, likely reflects the underlying severity of illness, the intrinsic mortality of several extra-cardiac conditions, and the high proportion of non-elective surgery, rather than prognostic discrimination by the adjudicated aetiology.

Management of myocardial injury remains largely empirical. One randomised trial demonstrated a reduction in major vascular complications in patients with MINS receiving 110 mg twice daily dabigatran, without a significant increase in major bleeding.^26^ More recently, an observational study reported that patients with PMI who were evaluated by a cardiologist had lower 1-yr mortality and fewer subsequent cardiovascular events.^27^ Although the mechanisms underlying this association remain uncertain, these findings suggest that outcomes after myocardial injury may be modifiable. However, intensification of β-blockers, statins, or antiplatelet therapy based solely on myocardial injury is not supported by robust evidence, and preoperative coronary revascularisation for stable symptoms in elective vascular surgery has not been shown to improve outcomes.^28^ Similarly, intraoperative haemodynamic strategies aimed at avoiding hypotension and hypertension have not consistently reduced complications despite observational links between hypotension, tachycardia, and MINS.^29^^,^^30^

### Limitations

This single-centre study has several limitations. First, postoperative troponin measurements were obtained either by institutional screening practice or at the discretion of the treating clinician. Screening practice reflected local implementation of the 2014 ESC guideline class IIb recommendation applicable during the study period, supported by institutional experience and published work. In addition, we deliberately retained patients with clinical signs warranting troponin measurement, as these may be relevant not to exclude, consistent with previous research.^12^ Nevertheless, this may have introduced selection bias, and therefore, our findings cannot be generalised to all surgical patients. Second, preoperative troponin concentrations were rarely available, precluding distinction between acute and chronic troponin elevation. Although sensitivity analysis excluding patients with chronic kidney disease partly addressed the potential contribution of chronic troponin elevation, dynamic troponin changes could not be assessed. Third, outcomes were limited to in-hospital mortality because follow-up after discharge was unavailable, which limits comparability with other studies and may underestimate the overall prognostic impact of different aetiologies. Fourth, postoperative hs-cTnT >50 ng L^−1^ was used as a dichotomous inclusion criterion based on previous work. Because data extraction only included troponin measurements exceeding this threshold, testing-rate distributions could not be described. Fifth, hierarchical adjudication has inherent limitations as it requires assignment of a single dominant cause, although multiple mechanisms may coexist. The framework was applied as closely as possible to a previously published aetiological model, with limited pragmatic adaptations to the available dataset. Therefore, it should be interpreted as a pragmatic descriptive approach, not as validation against, or comparison with, established definitions, assessment of prognostic discrimination, or proposal of a new classification system. Finally, moderate inter-rater agreement suggests limited reproducibility, which may constrain clinical applicability. This may reflect differences in reviewer experience or adherence to the adjudication protocol and co-occurring clinical conditions.

## Conclusion

In this single-centre cohort of 954 high-risk noncardiac surgical patients with postoperative hs-cTnT >50 ng L^−1^, myocardial injury was more often attributed to extra-cardiac than cardiac mechanisms, which were associated with higher in-hospital mortality. Future work should focus on the prognostic value of postoperative troponin elevation as a marker of perioperative risk across differing aetiologies.

## Authors’ contributions

Conceptualisation: BRBKS, SHE, FvL, Formal analysis: BRBKS, Methodology: BRBKS, SHE, FvL, Visualisation: BRBKS

Data curation: RvB, MS

Project administration: FvL

Investigation: RvB, MS

Supervision: RJS, SHE, FvL

Writing—original draft: BRBKS

Writing—review and editing: RJS, SHE, FvL

## Data availability statement

Data are available upon request of the corresponding author.

## Funding

Not applicable.

## Declaration of generative AI and AI-assisted technologies in the writing process

During the preparation of this work, the authors used ChatGPT during the writing process to improve readability and language. After using this service, the authors reviewed and edited the content as needed and take full responsibility for the content of the publication.

## Declarations of interest

The authors declare that they have no conflicts of interest.

## Figures and Tables

**Figure:**
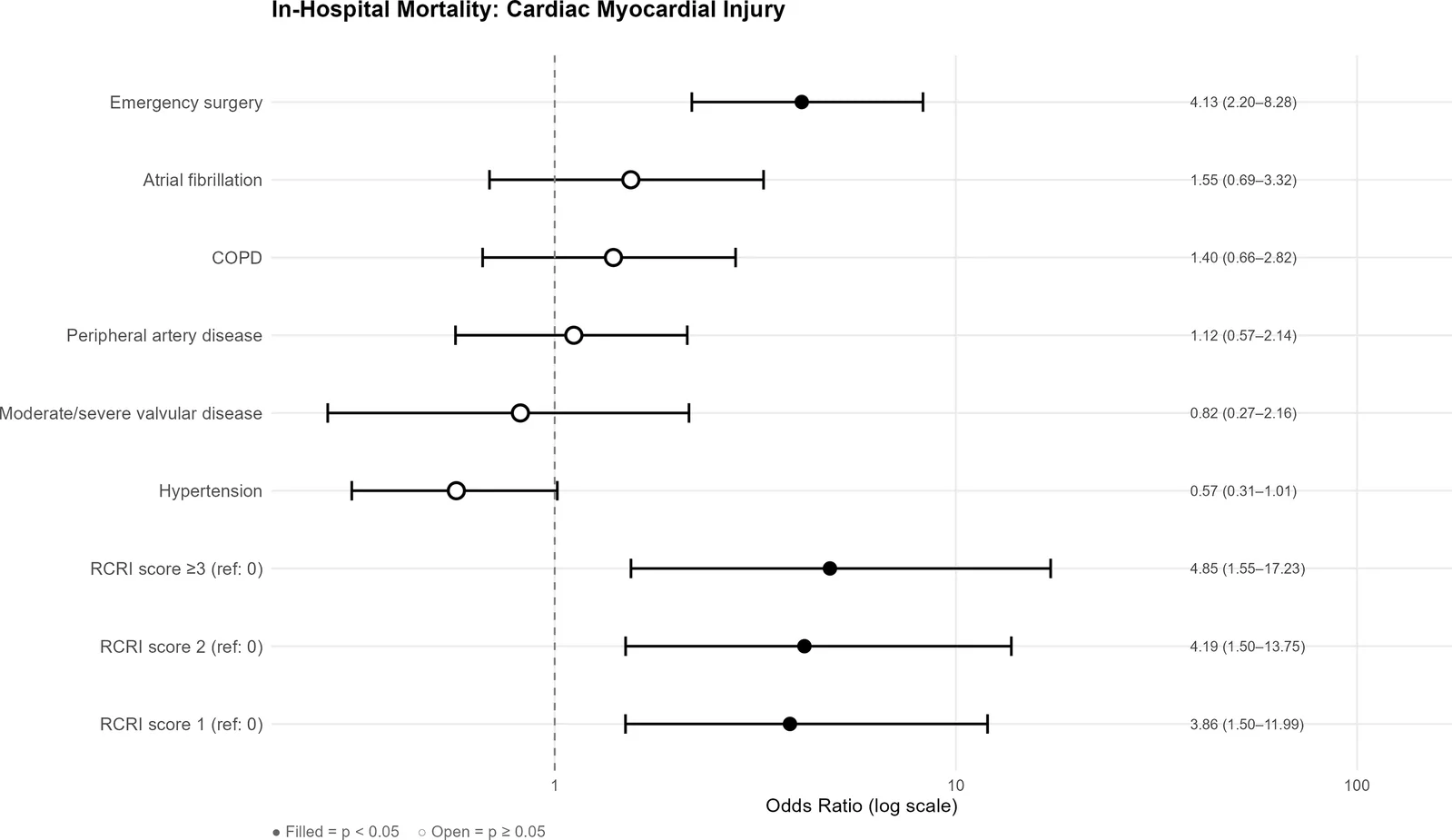


**Figure:**