# Supplementary material for: Aetiology and associated outcomes of myocardial injury as defined by postoperative troponin elevation in high-risk patients after noncardiac surgery: a single-centre retrospective cohort study
Source: BJA Open. 2026 Aug 13;19:100647. doi: 10.1016/j.bjao.2026.100647 (PMC13499167; doi:10.1016/j.bjao.2026.100647)
Supplement: Supplementary file 1 [file mmc1.docx]

Supplement 1. Aetiology definitions

| **Group** | **Aetiology** | **Operational definition** |
| --- | --- | --- |
| **Extra-cardiac** | Sepsis (SEPSIS-3) | Suspected infection with ≥2 qSOFA criteria: respiratory rate ≥22 min⁻¹, GCS <15, or systolic BP ≤100 mmHg or prolonged postoperative vasopressor use |
|  | Stroke | Ischaemic or haemorrhagic cerebrovascular event |
|  | Pulmonary embolism | Radiologically confirmed pulmonary embolism |
|  | Renal failure | Chronic or acute renal failure, including renal replacement therapy and/or eGFR <30 mL·min⁻¹·1.73 m⁻² |
|  | Cardiothoracic trauma | Blunt or penetrating cardiothoracic injury |
|  | Other | Extra-cardiac cause e.g. systemic inflammation without suspected infection |
| **Cardiac** | Type 1 myocardial infarction | Evidence of plaque rupture or culprit stenosis requiring revascularisation |
|  | Non-sinus tachycardia | Documented non-sinus tachyarrhythmia ≥120 beats·min⁻¹ |
|  | Acute heart failure | Acute heart failure requiring pharmacological and/or mechanical haemodynamic support including signs of congestion (dyspnoea, peripheral oedema, pleural effusion, rales), use of i.v. diuretics improving symptoms, without evidence of type 1 myocardial infarction |
|  | Type 2 myocardial injury with trigger | Intra- or postoperative documentation of hypotension (MAP < 65 mm Hg), anaemia (Hb < 6 mmol·L or < 9.7 g·dl⁻¹), hypoxaemia (defined as SpO2 < 88%), or sinus tachycardia ≥120 beats·min⁻¹ |
|  | Type 2 myocardial injury without trigger | No documentation of an identifiable trigger |
