# Supplementary material for: Aetiology and associated outcomes of myocardial injury as defined by postoperative troponin elevation in high-risk patients after noncardiac surgery: a single-centre retrospective cohort study
Source: BJA Open. 2026 Aug 13;19:100647. doi: 10.1016/j.bjao.2026.100647 (PMC13499167; doi:10.1016/j.bjao.2026.100647)
Supplement: Supplementary file 2 [file mmc2.docx]

Supplement 3

Sensitivity analysis excluding chronic kidney disease

|  | Main cohort (n = 954) | Non-CKD cohort (n = 714) |
| --- | --- | --- |
| **Classification** |  |  |
| Extra-cardiac | 544 (57%) | 379 (53%) |
| Cardiac | 410 (43%) | 335 (47%) |
|  |  |  |
| **In-hospital mortality** |  |  |
| Overall | 192 (20%) | 162 (23%) |
| Extra-cardiac | 129 (24%) | 112 (30%)* |
| Cardiac | 63 (15%) | 50 (15%)* |

CKD = chronic kidney disease; *extra-cardiac vs cardiac p-value <0.001

Sensitivity analysis of patients with hs-cTnT >50 ng·L⁻¹ measured at the EPC unit or after vascular surgery.

|  | Main cohort (n = 954) | Routinely sampled cohort (n = 502) |
| --- | --- | --- |
| **Classification** |  |  |
| Extra-cardiac | 544 (57%) | 263 (52%) |
| Cardiac | 410 (43%) | 239 (48%) |
|  |  |  |
| **In-hospital mortality** |  |  |
| Overall | 192 (20%) | 51 (10%) |
| Extra-cardiac | 129 (24%) | 31 (12%)* |
| Cardiac | 63 (15%) | 20 (8%)* |

* *P*-value = 0.26
